# Supplementary material for: Autophagy in High-Fat Diet and Streptozotocin-Induced Metabolic Cardiomyopathy: Mechanisms and Therapeutic Implications
Source: Int J Mol Sci. 2025 Feb 15;26(4):1668. doi: 10.3390/ijms26041668 (PMC11855906; doi:10.3390/ijms26041668)
Supplement: Supplementary file 1 [file ijms-26-01668-s001.zip › ijms-3406826-supplementary.pdf]

Table S1. Construction Methods for Obesity and Diabetes Models.

Models	Types of intervention		Duration	Organisms	Reference
Dietary Obesity and T2D models	HFD, 60% fat (Research Diets, D12492) (New Brunswick, NJ)		20 weeks	C57 mice	[31,32,39]
			24 weeks	Mito-Keima mice (FVB)	[24]
			12 weeks	C57, ApoE−/− mice Mito-Keima mice	[35,41,57]
			10 weeks	C57 mice	[59]
	HFD, 45% fat (Research Diets, D12451) (New Brunswick, NJ)		4 months	C57 mice	[33]
			5 months	FVB mice	[34]
			12 weeks	male mice	[60]
			20weeks	C57 mice	[36,60]
			8 weeks	WT mice	[38]
STZ -induced diabetic models	STZ, 30 mg/kg		once	C57 mice	[44]
	STZ, 50mg/kg/day		5 days	C57 mice	[47,49,50]
	STZ, 55 mg/kg/day		5 days	C57 mice	[45]
	STZ, 150 mg/kg		once	C57 mice	[51]
	HFD	STZ, 20 mg/kg/day	5days	C57 mice	[66]
		STZ, 25mg/kg/day	once	wistar rats	[58]
			Twice	SD rats	[48]
		STZ, 50 mg/kg/day	5 days	C57 mice	[52]
			5 days	SD rats	[46]
			5 days	Wistar rats	[53]
Genetic Obesity and diabetic models`	-		-	ob/ob mice	[29, 85]
	-		-	ZDF-Leprfa/fa rats	[28]
	HFD, 40% fat		10 weeks	KK-Ay mice	[40]
Vitro	high glucose		24h/48h/72 h	H9C2 cells	[45,50,51,58]
			24h/72 h	NRCMs	[53,54]
			48h	NMCMs	[47,49,65]
	palmitic acids		18 h	AC16 cells	[38]
			24 h	H9C2 cells	[32,50]
			15 min/12h	NRCMs	[24,70]
	high glucose +palmitic acids		24h	H9C2 cells	[50]
			48 h	NRCFs	[46]
